# Pro’s and con’s of the stepped wedge design in cluster randomised trials of quality improvement interventions: two current examples

**DOI:** 10.1186/1745-6215-14-S1-O87

**Published:** 2013-11-29

**Authors:** Tobias Dreischulte, Aileen Grant, Peter Donnan, Bruce Guthrie

**Affiliations:** 1 NHS Tayside, Dundee, UK; 2 University of Dundee, Dundee, UK

## 

The stepped wedge design, under which all trial participants receive the intervention but the order in which the intervention is received is randomised, is potentially useful to rigorously evaluate organisational interventions to improve quality and safety.

We use two examples of cluster-randomised stepped-wedge trials (DQIP and GP-POLY) to illustrate advantages and disadvantages of the design in evaluations of complex prescribing improvement interventions in primary care. DQIP is nearing completion and GP-POLY will start in 2013.

The intervention in both DQIP and GP-Poly involves outreach visits by researchers for education and informatics tool training, making sequential roll out a logistic necessity. The stepped wedge allows for this by design, but trial durations may be prolonged compared to parallel-arm trials and other designs, and arranging initiation visits to fit with randomisation schedules is challenging. Since all participants receive the intervention and there are multiple repeated measurements, practice sample size requirements in DQIP and GP-POLY were reduced compared to a parallel-arm design, but power calculations are more complex. Recruitment may be improved by offering the intervention to all participants, but creates potential problems for retention and avoiding contamination in practices with long lags between recruitment and intervention start. Because of the vulnerability of stepped wedge trials to time varying confounding, avoiding changes in intervention delivery to successive cohorts is important and needs careful planning.

The stepped wedge design is attractive for cluster randomised trials of quality improvement interventions, especially when staggering of intervention delivery is inevitable, but presents challenges for implementation that need careful planning.

